# Pediatrics

**Published:** 1899-03

**Authors:** Maud J. Frye

**Affiliations:** Clinical instructor in diseases of children, Medical Department, University of Buffalo


### PEDIATRICS.

REPORTED BY MAUD J. FRYE, M. D.,
Clinical instructor in diseases of children, Medical Department, University of Buffalo.

### THE URINE OF HEALTHY INFANTS AND CHILDREN.

CHURCHILL (*Archives of Pediatrics*, September, 1898,) reports the results of his investigations upon seventy infants and children ranging in age from one day to twelve years. Forty-eight were girls and twenty-two boys. One hundred and forty-six specimens were analysed and the following points noted: Total amount in twenty-four hours, color, reaction, specific gravity, presence or absence of albumin and sugar, percentage and total urea, percentages of phosphates, chlorides and sulphates. The urine was collected chiefly from children of the Half Orphan Asylum. The children were all healthy, robust and of the average weight. They were allowed to drink all the milk and water they wished.

The daily amount passed is low: at 4 years, 31 specimens, 299 c.c.; at 5 years, 17 specimens, 392 c.c.; at 8 years, 20 specimens, 628 c.c.; at 9 years, 25 specimens, 731 c.c.

The specific gravity in young infants is low, 10.01 to 10.05. In children of 1 year and upwards it is relatively high, in 129 of the examinations being 10.20 or over. The urea excretion, except in early infancy, is relatively higher than in adults, ranging in children above 1 year in age from 7.87 grms. at 3 years old to 23.21 at 12.

The chlorides were found quite constant at 11 per cent. up to 7 years, after which they were about 9 per cent. Purdy gives the percentage in adults as ranging from 16 to 18. The phosphates were found to be from 8 to 11 per cent. from 3 to 5 years; 5 to 7 per cent., from 6 to 12 years—the adult range being about 8 per cent. The percentage of sulphates was from 1 to 1.2, slightly higher than in adults. Neither albumin nor sugar was detected in any specimen. Nothing of interest was found in examination of the sediment. The reaction in all cases was acid. The color in most cases was pale.

### ACUTE APPENDICITIS IN CHILDREN.

EDWARD McGUIRE (*Richmond Jour. of Practice*, September, 1898,) says: Appendicitis before the age of four is very rare; after the tenth year, of frequent occurrence. The natural death-rate is higher than in adults, varying from 25 to 45 per cent. Many cases are overlooked or mistaken for colic, bilious attacks, and the like. The intensity of individual symptoms is more marked in children, but the symptoms as misleading as in adults. In the case reported in three days there were neither fever, pain nor tenderness, and the pulse was normal. The patient was thought to be convalescent. Reappearance of nausea and a complaint of pain, a little to the outside of the right nipple, were the first alarming symptoms. There were absolutely no local symptoms of appendicitis, no fever, and the pulse was 70. Guided, however, by the early history of the attack an operation was done, which revealed a gangrenous appendix, imbedded in a mass of infected adhesions.

The diagnosis in typical cases is based upon, first, the suddenness of the attack; second, the presence of abdominal pain; third, tenderness and muscular rigidity in the right iliac region; fourth, some nausea attending or following the pain, and in a large number of cases, some febrile disturbance.

Pain in the majority of cases is referred to the umbilical region or epigastrium. Afterward it becomes more centered in the right iliac region. It may be referred to any portion of the abdominal cavity, to the chest, or to the lumbar region. Pain may be violent or so slight as not to deter the child from play. Local tenderness in the right iliac fossa is almost pathognomonic. The area of tenderness, at first large, soon corresponds to McBurney's point. The situation of the tenderness is not constant, but varies with the position of the appendix. Tenderness may be absent.

Muscular rigidity is usually present. In the majority of cases it lessens or disappears in a day or two. The prominence of this symptom seems to have some relation to the proximity of the appendix to the anterior abdominal wall. Anesthesia will aid in distinguishing between muscular rigidity and tumor. Nausea is usually a more prominent symptom in childhood than in the adult, though not continuous. Acute nausea, occurring late in the disease, often marks the advent of perforation and the onset of septic peritonitis. Fever is no guide as to the seriousness of the disease; the pulse is a far better indication as to the gravity of the case. An acute peritonitis very rarely occurs in childhood from any other cause than appendicitis. While the large majority of cases are typical and easy of diagnosis, many are typical and perplexing to the diagnostician. In a large number of cases no amount of clinical experience or ability as a diagnostician will enable a surgeon to foretell the consequences or to recognise the true pathological conditions in a case of appendicitis. Regarding treatment it is to be said that simple cases have a low death-rate medically treated but a still lower one surgically. Grave cases have a high death-rate medically, but a low one surgically, if operation is not delayed. It is better to operate unnecessarily than unsuccessfully.

---

### HEATSTROKE IN INFANTS.

Snow (*Archives of Pediatrics*, October, 1898,) relates in detail the history of two cases. He says heatstroke in babies is easily confused with an acute gastro-enteric infection. The temperature is not often accurately taken or observed at all. The rapid collapse is ascribed to the very moderate vomiting and diarrhea. In typical heatstroke if the patient be thoroughly examined the whole attention of the physician will be centered in the extraordinary high temperature; the loose passages occur late, and are terminal symptoms; the cardiac depression and temperature, 107° to 108°, are not sufficiently accounted for by the usually moderate diarrhea; again, in an intestinal toxemia, the fever commonly subsides after vomiting and purging.

The therapeutics of insulation are well understood. In babies in addition to baths and packs, intestinal irrigations with cool water, 70° to 80°, are to be recommended. Subcutaneous injections of large quantities of artificial serum would probably revive the flagging strength of the heart, or would combat the unknown infection which, according to Sambon, is the cause of sunstroke.

